# Use of V(D)J recombination excision circles to identify T- and B-cell defects and to monitor the treatment in primary and acquired immunodeficiencies

**DOI:** 10.1186/1479-5876-11-119

**Published:** 2013-05-09

**Authors:** Federico Serana, Marco Chiarini, Cinzia Zanotti, Alessandra Sottini, Diego Bertoli, Andrea Bosio, Luigi Caimi, Luisa Imberti

**Affiliations:** 1 Inter-departmental AIL Laboratory, Diagnostics Department, Spedali Civili of Brescia, Brescia, Italy; 2 Clinical Biochemistry, Department of Molecular and Translational Medicine, University of Brescia, Brescia, Italy

**Keywords:** Immunodeficiency, T-cell receptor excision circles, K-deleting recombination excision circles, Newborn screening

## Abstract

T-cell receptor excision circles (TRECs) and kappa-deleting recombination excision circles (KRECs) are circular DNA segments generated in T and B cells during their maturation in the thymus and bone marrow. These circularized DNA elements persist in the cells, are unable to replicate, and are diluted as a result of cell division, thus are considered markers of new lymphocyte output. The quantification of TRECs and KRECs, which can be reliably performed using singleplex or duplex real-time quantitative PCR, provides novel information in the management of T- and B-cell immunity-related diseases. In primary immunodeficiencies, when combined with flow cytometric analysis of T- and B-cell subpopulations, the measure of TRECs and KRECs has contributed to an improved characterization of the diseases, to the identification of patients’ subgroups, and to the monitoring of stem cell transplantation and enzyme replacement therapy. For the same diseases, the TREC and KREC assays, introduced in the newborn screening program, allow early disease identification and may lead to discovery of new genetic defects. TREC and KREC levels can also been used as a surrogate marker of lymphocyte output in acquired immunodeficiencies. The low number of TRECs, which has in fact been extensively documented in untreated HIV-infected subjects, has been shown to increase following antiretroviral therapy. Differently, KREC number, which is in the normal range in these patients, has been shown to decrease following long-lasting therapy. Whether changes of KREC levels have relevance in the biology and in the clinical aspects of primary and acquired immunodeficiencies remains to be firmly established.

## Review

### V(D)J recombination and formation of excision circles

**Figure 1:**
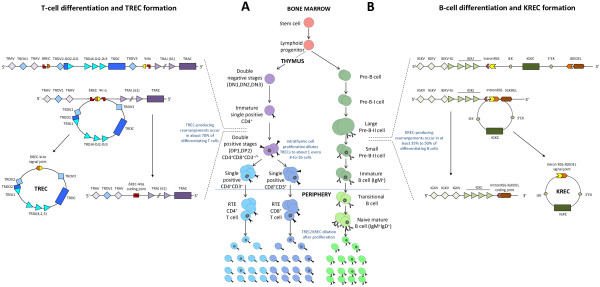
**New T- and B-cell generation. A**) Lymphoid progenitors migrate to the thymus, where they progressively rearrange T-cell receptor (*TR*) genes. Before the rearrangements of the *TR *alpha (*TRA*) locus, the *TR *delta (*TRD*) locus, which lies within the *TRA *locus, must be excised by DNA recombinations. Among them, the δREC–ψJα recombination, which occurs in about 70% of thymocytes [13], leads to the generation of a δREC–ψJα coding joint (CJ) in the chromosome and of a δREC–ψJα signal joint (SJ) in a circle of DNA called TR excision circle (TREC). Maturing thymocytes undergo 3–4 intrathymic divisions, in which TRECs cannot be duplicated; thus, only a fraction (about 1:8–1:16) of the originally TREC^+ ^cells will leave the thymus as TREC^+ ^recent-thymic emigrants (RTE) [6,13,15]. Peripheral proliferation will determine further TREC dilution. **B**) In the bone marrow, progenitor B cells undergo *V(D)J *rearrangements of the Ig heavy chain (*IGH*) locus followed by those of the light chains (*IGK *and *IGL*). After successful *IGH *rearrangements at the Pre-B stage, a *VJ* recombination on the *IGK *locus is initiated. If it is not productive, another recombination between the Ig kappa deleting element or like (IGKDEL) and one of the upstream recombination signal sequences (RSS) renders the *IGK *allele non-functional. In 30%-50% of cases, this occurs through the intronRSS-IGKDEL rearrangement, by which the *IGKC *exon and its enhancers (iEκ, 3’Eκ) are excised, with the creation of the so-called kappa-deleting recombination excision circles (KRECs). Thus, KRECs carry an intronRSS-IGKDEL SJ, and remain in the cells, but, as they cannot be replicated, they will be diluted during peripheral expansion of mature B cells. Instead, an intronRSS-IGKDEL CJ is formed and stably retained in the genomic DNA [7,8,10], because, due to the enhancer loss, any further rearrangement in the *IGK *locus is precluded.

Thymus and bone marrow (BM) are the primary anatomic sites for new T- and B-cell generation from undifferentiated hematopoietic precursors (Figure 1). Throughout this process, a highly heterogeneous lymphocyte repertoire is generated allowing the resulting cells to respond to a wide variety of antigenic stimuli [1-3]. Physiologically, T-cell maturation in the thymus progresses through distinct stages that are phenotypically defined by the expression of the T-cell receptor (TR) and the CD4 and CD8 co-receptors. On the basis of a programmed expression of distinct cell surface markers and of ordered gene rearrangements, thymocytes undergo different maturation steps before reaching the end stage [4]. B-lymphocyte development is divided into two main phases: an initial antigen-independent phase, in which precursor B cells mature into functional B lymphocytes in the BM, and an antigen-dependent phase, in which the mature B-cell compartment is maintained by regeneration, turnover and selection processes [5].

During the maturation processes of TR alpha/beta chains and of B-cell receptor (BcR) heavy and light chains, genomic rearrangements of antigen receptor genes generate functional receptors. This process is necessary because the gene complexes encoding the TR and BcR components do not contain a functional first exon, while including multiple variable (V), diversity (D), and joining (J) genes. In the antigen-independent differentiation phase, stepwise rearrangements are introduced into the genome to couple one of each segment together to form a functional first exon. The rearrangement of the TR alpha (*TRA*) genes has the peculiarity of involving the excision of delta-coding segments that, being nestled in the *TRA* locus between the *TRAV* and *TRAJ* genes, must be removed in order to allow the generation of the TRA chain. The excised DNA is circularized due to the ligation of the blunt DNA signal ends, thereby forming a signal joint (SJ) within the stable circular excision products termed TR excision circles (TRECs) [6]. Therefore, TRECs are the excised DNA circles formed during the process of TRA chain *VJ* recombination (Figure 1A). In B-cell maturation, K-deleting recombination excision circles (KRECs) are the products of recombination events determining the allelic and isotypic exclusion of the Ig kappa (*IGK*) locus [7-9]. They are created in those B lymphocytes that, after completing the IG heavy gene rearrangement, have failed to productively rearrange *IGK* genes on one or both alleles [10]. In these cells, the *IGK* locus becomes non-functional through the deletion of the *IGK* constant gene (*IGKC*) resulting from the recombination of the Ig kappa deleting element or like (IGKDEL), which is a sequence located approximately 24 kb downstream of the *IGKC*, with one of the upstream recombination signal sequences (RSS) located either at the 3’ side of a *IGK* variable gene segment (*IGKV*) or in the intron between the *IGK* joining segments (*IGKJ)* and the *IGKC*[10]. When the IGKDEL recombines with the RSS located in the *IGKJ-IGKC* intron (intronRSS), the formation of a coding joint (CJ) precludes any further rearrangements in the *IGK* locus. Thus, the CJ remains present in the genome, whereas an intronRSS-IGKDEL SJ is formed in the portion of DNA that is removed, thus forming the excision circle KREC (Figure 1B) [7,8].

### Quantification of TRECs and KRECs

Assays for quantification of TRECs and KRECs in peripheral blood are now performed in clinical and research laboratories to monitor naive T and B cells emigrating from the thymus and BM, respectively. Although TRECs are not markers of recent thymic emigrants by definition, because a small part remains present in peripheral blood in the so-called “old” thymic emigrants [6,11-14], several properties identify them as “bona fide” useful markers of thymic output. They are stable, do not replicate upon cell division and therefore are diluted in the progeny, do not degrade easily over time, and are (almost) exclusively of thymic origin, without extrathymic sources of *TR* rearrangements [6]. In particular, approximately 70% of T cells differentiating in the thymus contain TRECs [11], before being diluted 1:8–1:16 because of intrathymic divisions occurring during the last steps of maturation [13,15]. The TREC quantitative assay, initially proposed by Douek DC et al. [6], has been later modified in different ways and TREC number has been evaluated in different biological samples or calculated by different approaches, resulting into hardly comparable results. In fact, a more accurate TREC measurement has been obtained when quantification was performed relative to a control gene, such as chemokine (C-C motif) receptor 5, albumin, or *TRA* constant (*TRAC*) gene [12,16,17]. Furthermore, TREC content in peripheral blood has been reported either relative to peripheral blood mononuclear cells (PBMC), or quantified within sorted individual T-cell subsets [12,18]. In addition, the quantity of TRECs has been expressed either as absolute number of TREC molecules per μg of DNA within PBMC or T lymphocytes [16,19,20], or per 10^6^ cells, on the basis of the theoretical recovery of 1 μg of DNA from approximately 150,000 cells [21,22]. Because in adults’ samples TREC calculation per 10^6^ PBMC can lead to erroneous interpretations due to the diluting effect of peripheral T-cell divisions, this limitation has been, at least in part, overcome by expressing TRECs per ml of blood [23,24]. Finally, in spite of the present lack of information regarding TREC half-life [13], several mathematical models, which have taken into consideration confounders such as cell death, longevity of naive T cells, and intracellular degradation, have been recently proposed to identify the actual thymic output as a function of the TREC number [22,24].

Scanty information are presently available regarding the quantification of KRECs. Because the CJ remains in the genome, whereas the SJ present in the KREC is diluted after each cell division, the difference between the cycle threshold (Ct) values of the SJ and the CJ, obtained by real-time PCR, has been initially exploited to calculate the average number of divisions in a pool of B cells. In particular, because the intronRSS-IGKDEL rearrangement occurs late during BM differentiation, this measure is a good estimate of the average number of divisions performed by mature B cells after leaving the precursor B-cell compartment [7]. Furthermore, the frequency of B-cells that contain an intronRSS–IGKDEL rearrangement can be determined when a control gene is quantified together with the CJ [8]. A modified technique was used to determine the number of developing B lymphocytes in the BM of children with B-precursor acute lymphoblastic leukemia treated with allogeneic human stem-cell transplantation (HSCT) [25]. In this assay, KRECs were calculated by using the ΔCt method with a calibrator sample and by correcting the quantity and quality of DNA in the samples employing albumin as reference gene. The final number of KRECs relative to the calibrator was expressed as: 2^Ctcalibrator-Ctsample + log2(DNAconc. calibrator/DNAconc. sample)^.

More recently, we modified this newly proposed KREC assay and the widely used TREC assay by setting up a duplex quantitative real-time PCR that, by measuring together TRECs and KRECs, allows the quantification of newly produced T and B lymphocytes [26,27]. The main advantage of the combined assay is that the variability related to direct DNA quantification is eliminated by the use of a unique standard curve obtained by diluting the triple-insert plasmid, which contains TREC, KREC and *TRAC* fragments in a 1:1:1 ratio [26]. The last serves as a control for both the quality and quantity of genomic DNA in the sample. Furthermore, the simultaneous quantification of the two targets in the same reaction, results into a containment of laboratory costs. The duplex TREC/KREC assay is performed on DNA extracted from PBMC isolated from heparinized blood using primers and probes specific for TRECs, KRECs and *TRAC*. TRECs or KRECs per 10^6^ PBMC are calculated as the ratio between the mean number of TRECs or KRECs and the mean copy number of *TRAC* divided by 2, which is the number of *TRAC* copies per cell, and multiplied by 10^6^. This value, in conjunction with the combined lymphocyte - monocyte number in one ml of blood (which are the cells contained in a PBMC preparation), is used to calculate the number of TRECs or KRECs per ml of blood (TRECs or KRECs per 10^6^ PBMC) × (lymphocyte plus monocyte count in one ml of blood)/10^6^[26].

### TRECs and KRECs as markers of primary and acquired immunodeficiencies

TREC level has been extensively assayed in children with primary immunodeficiencies (PID) in whom the decreased number and function of T and/or B cells result into significant impairment of immunity. These rare, genetically determined disorders, which characteristically manifest during infancy and childhood with increased frequency of infections caused by unusual pathogens, are often accompanied by immunoregulatory defects [28]. Severe combined immunodeficiency (SCID), the most serious and lethal form of PID, is characterized by profound deficiencies of T- and B-cell functions and a low number of natural killer (NK) cells [29,30]. Infants with SCID develop failure to thrive, chronic diarrhea, and infections in the first few months of life. Graft-versus-host disease (GVHD), caused by maternal T-cell engraftment, may occur in these patients, who also show skin rash and organomegaly. All infants with the typical form of SCID, as well as those with another form of SCID, known as the Omenn syndrome, show extremely low or undetectable TREC levels (see Table 1, which completes and extends the data reported by van Zelm et al.) [8,26,31-42]. Despite the normal number of circulating T cells, abnormally low TREC levels were also observed in patients with zeta-chain (TCR) associated protein kinase 70kDa (ZAP70) deficiency and with the 22q11.2 deletion syndrome, especially in those without an identifiable thymus [31,38]. In contrast, patients with mutations in CD40 ligand, forkhead box protein 3 (*FOXP3*), and interleukin 10 receptor alpha chain, as well as patients with the p.R222C mutation in the interleukin 2 receptor gamma gene, display normal TREC levels [31]. As in SCID patients, the TREC assay contributed to understand the pathophysiology of other PID such as CD4^+^ T lymphocytopenia. In these patients, TREC levels correlated to the severity of the T-cell immunodeficiency [43]. Finally, TREC quantification has been used to improve our understanding of the T-cell abnormality that is sometimes observed in common variable immunodeficiency (CVID), although its precise role in this condition is still a matter of debate. While in fact some authors reported that the median TREC level in these patients was significantly higher than in healthy subjects, others found that this number was significantly reduced, in particular in patients characterized by a low number of switched/memory B cells (sIgD^-^CD27^+^), by a large proportion of CD19^hi^CD21^lo^ cells, and by an increased risk of autoimmunity and splenomegaly [41,44-46].

**Table 1:** T- and B-cell immunodeficiencies in which TRECs and KRECs have been evaluated

**Disease**	**Genetic defect**	**TRECs**	**KRECs**	**Author**
SCID	*ADA*	↓	nd	Roifman [31]; Gerstel-Thompson [32]; Morinishi [33]
	*AK2*	↓	↓	Borte [34]
	*CD3G*	↓	nd	Roifman [31]
	*CD8A*	↓	↓	Sottini [26]
	*IL2RA*	↓	nd	Roifman [31]
	*IL2RG*	↓	↑	Roifman [31]; Morinishi [33]; Borte [34]; Chan and Puck [35]
	*JAK3*	↓	↑	Morinishi [33]; Borte [34]; Chan and Puck [35]; Hale [36]
	*LIG4*	↓	nd	Roifman [31]; Morinishi [33]
	*PNP*	↓	nd	Gerstel-Thompson [32]
	*RAG1*	↓	↓	Sottini [26]; Roifman [31]; Morinishi [33]; Borte [34]
	*RAG2*	↓	nd	Roifman [31]; Morinishi [33]
	*RMRP*	↓	nd	Roifman [31]
	*ZAP70*	↓	nd	Roifman [31]
	*IL7RA*	↓	↑	Borte [34]
	unclassified	↓	↓	Roifman [31]; Borte [34]
	*CD40LG*	↑	↑	Sottini [26]; Roifman [31]; Borte [34]
	*FOXP3*	↑	nd	Roifman [31]
	*IL10RA*	↑	nd	Roifman [31]
	*IL2RG* (p.R222C mutation)	↑	nd	Roifman [31]
	partial *ADA*	↑	nd	Roifman [31]
DGS	22q11.2 deletion	↓	nd	Routes [37]; Lima [38]
NBS	*NBN*	↑	↓	Borte [34]; van der Burg [39]
XLA	*BTK*	↑	↓	Borte [34]; Nakagawa [40]
non-XLA		nd	↑	Nakagawa [40]
AT	*ATM*	borderline	↓	Borte [34]
WAS	*WAS*	↓	↓	Sottini [26]
CVID (adults)		↓	↓	Serana [41]
CVID (newborn)		↑	↑	Borte [34]; Kamae [42]
IgAD		↑	↑	Borte [34]

*ADA*: adenosine deaminase; *AK2*: adenylate kinase 2; *AT*: ataxia-telangectasia; *ATM*: AT mutated; *BTK*: Bruton agammaglobulinemia tyrosine kinase; *CD3G*: CD3g molecule, gamma (CD3-TCR complex); *CD40LG*: CD40 ligand; *CD8A*: CD8A molecule; CVID: common variable immunodeficiency; DGS: DiGeorge syndrome; *FOXP3*: forkhead box P3; IgAD: IgA deficiency; *IL10RA*: interleukin 10 receptor, alpha; *IL2RA*: interleukin 2 receptor, alpha; *IL2RG*: interleukin 2 receptor, gamma; *IL7RA*: interleukin 7 receptor, alpha; *JAK3*: Janus kinase 3; KRECs: k-deleting recombination excision circles; *LIG4*: ligase IV, DNA, ATP-dependent; *NBN*: nibrin; NBS: Nijmegen breakage syndrome; nd: not done; *PNP*: purine nucleoside phosphorylase; *RAG1*: recombination activating gene 1; *RAG2*: recombination activating gene 2; *RMRP*: RNA component of mitochondrial RNA processing endoribonuclease; SCID: severe combined immunodeficiency; TRECs: T-cell receptor excision circle; *WAS*: Wiskott-Aldrich syndrome; XLA: X-linked agammaglobulinemia; *ZAP70*: zeta-chain (TCR) associated protein kinase 70kDa.

↑: over the cut-off; ↓: lower than the cut-off or undetectable.

KREC level has been evaluated only very recently in children with PID, and mainly in patients affected by X-linked or non X-linked agammaglobulinemia (XLA and non-XLA, respectively), and in those with CVID. While XLA, the most severe form of B-cell defects, results from a mutation in the Bruton agammaglobulinemia tyrosine kinase (*BTK)* gene that causes a B-cell differentiation arrest in the BM, with consequent absence of mature B cells and serum Ig, non-XLA is characterized by hypogammaglobulinemia with decreased B-cell counts in the absence of the *BTK* gene mutation [47]. In both forms, which account for about 20% of all B-cell defects, recurrent infections appear between 3 and 18 months of age [48]. KREC measurement has been proposed as a potential tool for the identification of these two diseases, because B-cell maturation defects occur before IGKDEL events, and therefore KRECs should be not produced/detected in these patients. Indeed, a study performed by analyzing KRECs derived from dried blood spots revealed that no KRECs were detected in 30 XLA and 5 non-XLA patients [40]. We employed the combined TREC/KREC assay to measure the extent of T- and B-cell neoproduction in CVID adult patients with no acute infections and undergoing Ig passive immunotherapy. The number of TREC^+^ lymphocytes, which depended on age and gender, was significantly reduced. At the same time, KREC number was lower than in controls, but it did not change with age and was not influenced by the gender. Of note, 35% of CVID patients had less KRECs/ml than the 5^th^ percentile calculated in controls [41]. On the contrary, newborns affected by CVID (and those with some other typical SCID, see Table 1) displayed a number of KRECs comparable to that of healthy newborns [34]. Very recently, Kamae et al. [42] showed that the amount of TRECs and KRECs can be a useful marker to assess the pathogenesis and clinical severity of CVID in distinct patients.

While the quantification of TRECs has been performed for years and is used as a surrogate marker of thymic output in HIV-infected patients [6], the precise interpretation of TREC data obtained in acquired immunodeficiency subjects is more challenging. Indeed, it depends heavily on the assumed fate of naive T cells, recent thymic emigrants in particular [12,24,49], and on the different clinical-pathological parameters of infected subjects, such as age and plasma HIV-1 RNA. Although several studies have shown that HIV-infected subjects have a lower number of TRECs than that found in age-matched uninfected individuals [6,12,16,50], the TREC number is variable among patients and overlaps with that of controls [16]. Using the TREC/KREC assay we found that the number of TREC^+^ cells of patients that need antiretroviral therapy was significantly lower not only when compared to controls, but also when compared to HIV-infected patients with a relatively conserved CD4 cell number and not requiring treatment according to the current guidelines. In this latter group, the number of KRECs was significantly higher than that found in patients needing treatment, but similar to that found in age-matched healthy controls [51].

### TREC/KREC assay for detection of neonatal primary immunodeficiency diseases

Early recognition of SCID should be considered a pediatric emergency, because a diagnosis before the vaccination programs and the onset of recurrent infections allows lifesaving HSCT, enzyme replacement, or gene therapy [52-60]. Similarly, early diagnosis and treatment, including periodical intravenous Ig replacement therapy, are essential to improve the prognosis and the quality of life of patients with B-cell defects. However, infants with SCID or agammaglobulinemia often appear normal at birth, have no family history of immunodeficiency [61], and, consequently, many of them are not identified until life-threatening infections occur.

The demonstration that the TREC assay detects SCID patients regardless of the underlying genetic defects [33] and that agammaglobulinemia patients can be identified with KREC quantification [40], suggested that the TREC and KREC assays can be used for the detection of SCID and agammaglobulinemia in newborn screening (NBS) programs. This is particularly true for SCID, which satisfies the criteria recommended by the Secretary’s Advisory Committee on Heritable Disorders in Newborns and Children [62]. The TREC assay was the first to be modified and calibrated to allow the test to be performed on DNA extracted from small spots of dried blood, to be highly sensitive and specific for SCID, cost-effective, reproducible and, therefore, amenable to be used in a population-based screening [63]. Pilot studies of NBS for SCID, integrated with diagnosis and management guidelines, have been performed in some U.S. states [61]. Accordingly, in 2008, Wisconsin became the first state to implement mandatory NBS for T-cell deficiencies [37], followed, in February 2009, by Massachusetts [32,64] and, later, by New York, Navajo Nation, California, Puerto Rico and Louisiana [65]. By using in-house modifications of the TREC assay (with the exception of California, where a TREC assay kit under development by a company was used), these pilot studies identified, among the 961,925 newborns screened, several cases of T-cell deficits, 14 of which resulted to be SCID at the confirmatory tests, 6 SCID variants, and 40 T-cell lymphopenia not related to SCID [32,35,63-65]. All infants with SCID identified in Wisconsin and Massachusetts have undergone transplantation or enzyme replacement therapy and are all alive [36,37]. More recently, also the TREC/KREC assay was modified, making it suitable to be performed on a single Guthrie card punch. This assay has been validated in a cohort of 2,560 NBS cards from healthy neonates and used to identify patients with SCID, XLA, ataxia-telangiectasia, and Nijmegen-breakage-syndrome [34]. The early diagnosis of children with XLA is expected to significantly improve their quality of life and contribute to a reduction of health care costs. Indeed, because their clinical phenotype is less severe than that in SCID, the risk of undiagnosed disease in their first years of life in absence of a screening program would foster the development of more severe organ damage.

Conclusively, considering that the TREC measurement is performed at the relatively low cost of approximately $ 5.50 per assay [37], that the inclusion of KREC detection in the TREC assay barely increases the costs, and that SCID and agammaglobulinemia have a combined estimated incidence of 1:30,000-1:50,000 births, a single assay capable of screening for both conditions should further improve the cost-effectiveness of the NBS for PID. The importance of introducing PID in NBS programs has been underlined by the guidelines very recently proposed by Borte et al. [66].

### Quantification of TRECs and KRECs for therapy monitoring of primary and acquired immunodeficiencies

Patients with SCID have a very short life-expectancy if they are not promptly treated with HLA-identical or HLA-haploidentical HSCT [67,68], which frequently results into a durable engraftment of all hematopoietic cell lineages that leads to a restoration of immunological functions in the absence of GVHD [68,69]. Therefore, the success of HSCT mainly depends on the rate of cellular immune reconstitution [70-75]. However, while innate immunity shows a full phenotypic and functional recovery within a month from transplantation [76], the recovery of T cells can be significantly delayed, even in comparison to that of B cells, which can occur within 6–9 months (although a fully functional reconstitution encompassing the synthesis of all Ig isotypes may require up to 2 years) [76-80]. Therefore*,* due to the impairment of both T- and B-cell functions, HSCT recipients are more prone to infections and relapse of malignancies [81,82], and the risk of developing these complications notably correlates with the recovery of CD4^+^ T cells [83]. Because it is known that, in these patients, the appearance of TRECs is the most predictive indicator for long-term T-cell reconstitution [84], a frequent monitoring of the T-cell immunity and TREC number after HSCT can help identify those patients who will eventually fail to be properly reconstituted, thus requiring additional therapies that could be more timely initiated. The use of the TREC assay may be also relevant to verify the outcome of unconditioned transplantations with matched sibling and family donors. In these cases, which are highly successful in terms of survival outcome, the assay may be informative in detecting a long-term thymopoiesis, or to establish whether the T-cell reconstitution is due to an engraftment of mature T cells that will ultimately be exhausted, thus leading to a long-term T-cell deficiency [83]. Indeed, a low TREC number has been found after unconditioned procedures, indicating the occurrence of only a limited prethymic progenitor-cell engraftment. Therefore, in these transplantations, the long term T-cell immunity is likely to be sustained by a pool of mature T cells [85]. Finally, the progressive decline of thymopoiesis observed after HSCT has been attributed to a number of factors. These include: a defective thymic microenvironment, which is derived from pre-existing alterations or GVHD-induced damages; the lack of an adequate reservoir of healthy donor stem cells (associated with the lack of conditioning); an insufficient HSC dose; or the recipient’s age [83,86,87].

The long-term (functional) immune recovery was particularly difficult to assess in Adenosine deaminase (ADA)-SCID patients. Treatments of these patients have included HSCT from an HLA-identical sibling donor without conditioning regimen, when available [88], enzyme replacement therapy [89], transplantation with unrelated donor cells [88], and gene therapy [90,91]. Using the TREC/KREC assay, we conducted a detailed evaluation of T- and B-cell reconstitution in patients with broadly overlapping immunologic parameters, treated with HSCT or with polyethylene glycol-conjugated (PEG)-ADA. We found that the TREC level rose in both groups, but then quickly declined and persisted at low levels in the PEG-ADA group. The B-cell generation, studied both by B-cell subset phenotyping and KREC quantification, was more often impaired in the PEG-ADA group, and homeostatic proliferation could only partly compensate the decreased BM output [27]. 

TRECs/KRECs were also measured in patients with other PID (SCID T^-^B^-^NK^+^, SCID T^-^B^-^NK^-^, SCID T^-^B^+^NK^+^, SCID T^-^B^+^NK^-^, X-linked hyper IgM, Wiskott-Aldrich syndrome and familial hemophagocytic lymphohistiocytosis) who underwent HSCT and were followed up for a period ranging from 12 to 79 months. These children were heterogeneous with respect to sex, immunodeficiency type, graft donor, age of the donors and recipients of HSCT, type of conditioning, and occurrence and grade of GVHD. We found that the post-transplantation increase of TRECs and KRECs could be either strictly associated or independent of each other, and it was followed by the normalization of the T-cell repertoire and Ig production. Some patients showed a sharp, but transitory increase in new lymphocyte output which declined later on; in other patients, the TREC and KREC number remained very low for the entire period of surveillance [26].

An early output of B and T lymphocytes from the production and maturation sites, which is indicative of successful clinical outcome, was demonstrated, using a similar approach, also in transplanted SCID patients with a deficient recombination-activating gene 2 [92].

The TREC assay was also extensively used to evaluate the immune reconstitution in HIV-infected patients treated with antiviral therapy [6,93-96]. Common knowledge is that thymic immune reconstitution of HIV-infected patients is more successful in children than in adults, suggesting that increased thymic output could play, at least in the formers, a predominant role in immune recovery [97]. TREC number increases in both virological responder and non-responder children, indicating that the persistence of viremia during therapy does not impair the increase in thymic function and the subsequent output of naive cells [98]. In adult patients with HIV-related lymphoma highly responder to antiretroviral therapy, the analysis of the kinetics and the extent of T-cell reconstitution before and after HSCT demonstrated that the very low level of TRECs before transplantation significantly increased following treatment [99], due to an extent of immune recovery close to that observed in transplanted HIV-negative patients with lymphoma [100]. Similarly, TREC monitoring in HIV-infected patients, long-term treated with low-doses of recombinant human growth hormone therapy, showed a recovery of thymopoiesis, as confirmed also by thymic index, density and area quantification [101]. Only few data are available on the number of KRECs in treated HIV-infected patients. One year of therapy did not modify the KREC number, while the long-lasting treatment (6 years) resulted in a significant decrease in new B-cell release from the BM [51]. However, due to the scarcity of available data, the usefulness of KREC quantification in these patients remains to be firmly established.

### Limitations of the use TRECs and KRECs as immunological markers of immunodeficiency

Because TRECs and KRECs are not produced if maturation of T cells and B cells ceases at early steps, they are diluted out after cell division, they can persist in old thymic emigrant cells, and they disappear after cell death, caution is warranted in interpreting their number in the clinical setting. Therefore, even though expressing the amount of TRECs or KRECs per ml of blood overcomes the issue of peripheral dilution [24,102], this measurement alone cannot still be considered a direct clinical marker of immune disease. Indeed, TRECs can be decreased in subjects that are not immunodeficient, such as premature babies and Down syndrome patients [103,104]. Therefore, it must be also emphasized that TREC/KREC determination is only the first-tier assay, that must be followed by appropriate second-tier assays defining the disease (if any) that results in the low TREC/KREC values.

## Conclusions

The increasing laboratory and clinical evidences reported in this review, which extends and completes the recent one by van Zelm et al. [8], indicate that the quantification of TRECs and KRECs can be very informative in the management of patients with primary and acquired immunodeficiencies. It appeared that it is technically feasible to introduce the TREC/KREC assay into routine laboratory practice both for NBS and for a more critical monitoring of the rate of T- and B-cell immune reconstitution following HSCT and antiretroviral therapy.

## Abbreviations

ADA: Adenosine deaminase; BcR: B-cell receptor; BM: bone marrow; BTK: Bruton agammaglobulinemia tyrosine kinase; CJ: Coding joint; Ct: Cycle threshold; CVID: common variable immunodeficiency; D: Diversity; FOXP3: Forkhead box protein 3; GVHD: Graft-versus-host disease; HSCT: Human stem-cell transplantation; IGK: Ig kappa; IGKC: IGK constant gene; IGKDEL: IGK deleting element or like; IGKJ: IGK joining genes; J: Joining; KRECs: Kappa-deleting recombination excision circles; NBS: Newborn screening; NK: Natural killer; PBMC: Peripheral blood mononuclear cells; PEG: Polyethylene glycol; PID: Primary immunodeficiencies; RSS: Recombination signal sequences; SCID: Severe combined immunodeficiency; SJ: Signal joint; TR: T-cell receptor; TRA: TR alpha; TRAC: TRA constant gene; TRECs: T-cell receptor excision circles; V: Variable; XLA: X-linked agammaglobulinemia; ZAP70: zeta-chain (TCR) associated protein kinase 70kDa.

## Competing interests

The authors declare that they have no conflicts of interest.

## Authors’ contributions

FS discussed the outline and contributed to the drafting of the article, MC, CZ, AS, DB, AB assisted in drafting the manuscript and approved the final version; LC discussed the manuscript and approved the final version; and LI wrote the manuscript. All authors read and approved the final manuscript.

## References

[B1] Nossal GJ Negative selection of lymphocytes Cell 1994 76 229 239 Review 10.1016/0092-8674(94)90331-X 8293461

[B2] Fry AM Jones LA Kruisbeek AM Matis LA Thymic requirement for clonal deletion during T cell development Science 1989 246 1044 1046 10.1126/science.2511630 2511630

[B3] Hodes RJ Sharrow SO Solomon A Failure of T cell receptor V beta negative selection in an athymic environment Science 1989 246 1041 1044 10.1126/science.2587987 2587987

[B4] Dik WA Pike-Overzet K Weerkamp F de Ridder D de Haas EF Baert MR van der Spek P Koster EE Reinders MJ van Dongen JJ Langerak AW Staal FJ New insights on human T cell development by quantitative T cell receptor gene rearrangement studies and gene expression profiling J Exp Med 2005 201 1715 1723 10.1084/jem.20042524 15928199 PMC2213269

[B5] Ghia P Ten Boekel E Rolink AG Melchers F B-cell development: a comparison between mouse and man Immunol Today 1998 19 480 485 Review 10.1016/S0167-5699(98)01330-9 9785673

[B6] Douek DC McFarland RD Keiser PH Gage EA Massey JM Haynes BF Polis MA Haase AT Feinberg MB Sullivan JL Jamieson BD Zack JA Picker LJ Koup RA Changes in thymic function with age and during the treatment of HIV infection Nature 1998 396 690 695 10.1038/25374 9872319

[B7] van Zelm MC Szczepanski T van der Burg M van Dongen JJ Replication history of B lymphocytes reveals homeostatic proliferation and extensive antigen-induced B cell expansion J Exp Med 2007 204 645 655 10.1084/jem.20060964 17312005 PMC2137914

[B8] van Zelm MC van der Burg M Langerak AW van Dongen JJ PID comes full circle: applications of V(D)J recombination excision circles in research, diagnostics and newborn screening of primary immunodeficiency disorders Front Immunol 2011 2 12 22566803 10.3389/fimmu.2011.00012 PMC3342366

[B9] Siminovitch KA Bakhshi A Goldman P Korsmeyer SJ A uniform deleting element mediates the loss of kappa genes in human B cells Nature 1985 316 260 262 10.1038/316260a0 3927169

[B10] Beishuizen A de Bruijn MA Pongers-Willemse MJ Verhoeven MA van Wering ER Hählen K Breit TM de Bruin-Versteeg S Hooijkaas H van Dongen JJ Heterogeneity in junctional regions of immunoglobulin kappa deleting element rearrangements in B cell leukemias: a new molecular target for detection of minimal residual disease Leukemia 1997 11 2200 2207 10.1038/sj.leu.2400904 9447841

[B11] Verschuren MC Wolvers-Tettero IL Breit TM Noordzij J van Wering ER van Dongen JJ Preferential rearrangements of the T cell receptor-delta-deleting elements in human T cells J Immunol 1997 158 1208 1216 9013961

[B12] Hazenberg MD Otto SA Cohen Stuart JW Verschuren MC Borleffs JC Boucher CA Coutinho RA Lange JM Rinke de Wit TF Tsegaye A van Dongen JJ Hamann D de Boer RJ Miedema F Increased cell division but not thymic dysfunction rapidly affects the T-cell receptor excision circle content of the naive T cell population in HIV-1 infection Nat Med 2000 6 1036 1042 10.1038/79549 10973325

[B13] Hazenberg MD Verschuren MC Hamann D Miedema F van Dongen JJ T cell receptor excision circles as markers for recent thymic emigrants: basic aspects, technical approach, and guidelines for interpretation J Mol Med 2001 79 631 640 Review 10.1007/s001090100271 11715066

[B14] Sodora DL Douek DC Silvestri G Montgomery L Rosenzweig M Igarashi T Bernacky B Johnson RP Feinberg MB Martin MA Koup RA Quantification of thymic function by measuring T cell receptor excision circles within peripheral blood and lymphoid tissues in monkeys Eur J Immunol 2000 30 1145 1153 10.1002/(SICI)1521-4141(200004)30:4<1145::AID-IMMU1145>3.0.CO;2-7 10760804

[B15] van der Weerd K Dik WA Schrijver B Bogers AJ Maat AP van Nederveen FH van Hagen PM van Dongen JJ Langerak AW Staal FJ Combined TCRG and TCRA TREC analysis reveals increased peripheral T-lymphocyte but constant intra-thymic proliferative history upon ageing Mol Immunol 2013 53 302 312 10.1016/j.molimm.2012.08.019 23000520

[B16] Zhang L Lewin SR Markowitz M Lin HH Skulsky E Karanicolas R He Y Jin X Tuttleton S Vesanen M Spiegel H Kost R van Lunzen J Stellbrink HJ Wolinsky S Borkowsky W Palumbo P Kostrikis LG Ho DD Measuring recent thymic emigrants in blood of normal and HIV-1-infected individuals before and after effective therapy J Exp Med 1999 190 725 732 10.1084/jem.190.5.725 10477556 PMC2195623

[B17] Zubakov D Liu F van Zelm MC Vermeulen J Oostra BA van Duijn CM Driessen GJ van Dongen JJ Kayser M Langerak AW Estimating human age from T-cell DNA rearrangements Curr Biol 2010 20 R970 R971 10.1016/j.cub.2010.10.022 21093786

[B18] Ponchel F Toomes C Bransfield K Leong FT Douglas SH Field SL Bell SM Combaret V Puisieux A Mighell AJ Robinson PA Inglehearn CF Isaacs JD Markham AF Real-time PCR based on SYBR-green I fluorescence: an alternative to the TaqMan assay for a relative quantification of gene rearrangements, gene amplifications and micro gene deletions BMC Biotechnol 2003 3 18 10.1186/1472-6750-3-18 14552656 PMC270040

[B19] Nobile M Correa R Borghans JA D'Agostino C Schneider P De Boer RJ Pantaleo G Swiss HIV Cohort Study De novo T-cell generation in patients at different ages and stages of HIV-1 disease Blood 2004 104 470 477 10.1182/blood-2003-12-4265 15059846

[B20] Hug A Korporal M Schröder I Haas J Glatz K Storch-Hagenlocher B Wildemann B Thymic export function and T cell homeostasis in patients with relapsing remitting multiple sclerosis J Immunol 2003 171 432 437 12817027 10.4049/jimmunol.171.1.432

[B21] Hazenberg MD Otto SA de Pauw ES Roelofs H Fibbe WE Hamann D Miedema F T-cell receptor excision circle and T-cell dynamics after allogeneic stem cell transplantation are related to clinical events Blood 2002 99 3449 3453 10.1182/blood.V99.9.3449 11964316

[B22] Bains I Thiébaut R Yates AJ Callard R Quantifying thymic export: combining models of naive T cell proliferation and TCR excision circle dynamics gives an explicit measure of thymic output J Immunol 2009 183 4329 4336 10.4049/jimmunol.0900743 19734223

[B23] Krenger W Schmidlin H Cavadini G Holländer GA On the relevance of TCR rearrangement circles as molecular markers for thymic output during experimental graft-versus-host disease J Immunol 2004 172 7359 7567 15187112 10.4049/jimmunol.172.12.7359

[B24] Ribeiro RM Perelson AS Determining thymic output quantitatively: using models to interpret experimental T-cell receptor excision circle (TREC) data Immunol Rev 2007 216 21 34 Review 17367332 10.1111/j.1600-065X.2006.00493.x

[B25] Fronkova E Muzikova K Mejstrikova E Kovac M Formankova R Sedlacek P Hrusak O Stary J Trka J B-cell reconstitution after allogeneic SCT impairs minimal residual disease monitoring in children with ALL Bone Marrow Transplant 2008 42 187 196 10.1038/bmt.2008.122 18490915

[B26] Sottini A Ghidini C Zanotti C Chiarini M Caimi L Lanfranchi A Moratto D Porta F Imberti L Simultaneous quantification of recent thymic T-cell and bone marrow B-cell emigrants in patients with primary immunodeficiency undergone to stem cell transplantation Clin Immunol 2010 136 217 227 10.1016/j.clim.2010.04.005 20452829

[B27] Serana F Sottini A Chiarini M Zanotti C Ghidini C Lanfranchi A Notarangelo LD Caimi L Imberti L The different extent of B and T cell immune reconstitution after hematopoietic stem cell transplantation and enzyme replacement therapies in SCID patients with adenosine deaminase deficiency J Immunol 2010 185 7713 7722 10.4049/jimmunol.1001770 21057082

[B28] Notarangelo LD Primary immunodeficiencies J Allergy Clin Immunol 2010 125 2 Suppl 2 S182 S194 20042228 10.1016/j.jaci.2009.07.053

[B29] Buckley RH Molecular defects in human severe combined immunodeficiency and approaches to immune reconstitution Annu Rev Immunol 2004 22 625 655 Review 10.1146/annurev.immunol.22.012703.104614 15032591

[B30] Fischer A Le Deist F Hacein-Bey-Abina S André-Schmutz I Basile Gde S de Villartay JP Cavazzana-Calvo M Severe combined immunodeficiency. A model disease for molecular immunology and therapy Immunol Rev 2005 203 98 109 Review 10.1111/j.0105-2896.2005.00223.x 15661024

[B31] Roifman CM Somech R Kavadas F Pires L Nahum A Dalal I Grunebaum E Defining combined immunodeficiency J Allergy Clin Immunol 2012 130 177 183 10.1016/j.jaci.2012.04.029 22664165

[B32] Gerstel-Thompson JL Wilkey JF Baptiste JC Navas JS Pai SY Pass KA Eaton RB Comeau AM High-throughput multiplexed T-cell-receptor excision circle quantitative PCR assay with internal controls for detection of severe combined immunodeficiency in population-based newborn screening Clin Chem 2010 56 1466 1474 10.1373/clinchem.2010.144915 20660142

[B33] Morinishi Y Imai K Nakagawa N Sato H Horiuchi K Ohtsuka Y Kaneda Y Taga T Hisakawa H Miyaji R Endo M Oh-Ishi T Kamachi Y Akahane K Kobayashi C Tsuchida M Morio T Sasahara Y Kumaki S Ishigaki K Yoshida M Urabe T Kobayashi N Okimoto Y Reichenbach J Hashii Y Tsuji Y Kogawa K Yamaguchi S Kanegane H Miyawaki T Yamada M Ariga T Nonoyama S Identification of severe combined immunodeficiency by T-cell receptor excision circles quantification using neonatal Guthrie cards J Pediatr 2009 155 829 833 10.1016/j.jpeds.2009.05.026 19628217

[B34] Borte S von Döbeln U Fasth A Wang N Janzi M Winiarski J Sack U Pan-Hammarström Q Borte M Hammarström L Neonatal screening for severe primary immunodeficiency diseases using high-throughput triplex real-time PCR Blood 2012 119 2552 2555 10.1182/blood-2011-08-371021 22130802

[B35] Chan K Puck JM Development of population-based newborn screening for severe combined immunodeficiency J Allergy Clin Immunol 2005 115 391 398 10.1016/j.jaci.2004.10.012 15696101

[B36] Hale JE Bonilla FA Pai SY Gerstel-Thompson JL Notarangelo LD Eaton RB Comeau AM Identification of an infant with severe combined immunodeficiency by newborn screening J Allergy Clin Immunol 2010 126 1073 1074 10.1016/j.jaci.2010.08.043 20933257

[B37] Routes JM Grossman WJ Verbsky J Laessig RH Hoffman GL Brokopp CD Baker MW Statewide newborn screening for severe T-cell lymphopenia JAMA 2009 302 2465 2470 10.1001/jama.2009.1806 19996402

[B38] Lima K Abrahamsen TG Foelling I Natvig S Ryder LP Olaussen RW Low thymic output in the 22q11.2 Deletion syndrome measured by CCR9+CD45RA+ T cell counts and T cell receptor rearrangement excision circles Clin Exp Immunol 2010 161 98 107 20491792 10.1111/j.1365-2249.2010.04152.x PMC2940154

[B39] van der Burg M Pac M Berkowska MA Goryluk-Kozakiewicz B Wakulinska A Dembowska-Baginska B Gregorek H Barendregt BH Krajewska-Walasek M Bernatowska E van Dongen JJ Chrzanowska KH Langerak AW Loss of juxtaposition of RAG-induced immunoglobulin DNA ends is implicated in the precursor B-cell differentiation defect in NBS patients Blood 2010 115 4770 4777 10.1182/blood-2009-10-250514 20378756

[B40] Nakagawa N Imai K Kanegane H Sato H Yamada M Kondoh K Okada S Kobayashi M Agematsu K Takada H Mitsuiki N Oshima K Ohara O Suri D Rawat A Singh S Pan-Hammarström Q Hammarström L Reichenbach J Seger R Ariga T Hara T Miyawaki T Nonoyama S Quantification of κ-deleting recombination excision circles in Guthrie cards for the identification of early B-cell maturation defects J Allergy Clin Immunol 2011 128 223 225.e2 10.1016/j.jaci.2011.01.052 21397315

[B41] Serana F Airò P Chiarini M Zanotti C Scarsi M Frassi M Lougaris V Plebani A Caimi L Imberti L Thymic and bone marrow output in patients with common variable immunodeficiency J Clin Immunol 2011 31 540 549 10.1007/s10875-011-9526-6 21491094

[B42] Kamae C Nakagawa N Sato H Honma K Mitsuiki N Ohara O Kanegane H Pasic S Pan-Hammarström Q van Zelm MC Morio T Imai K Nonoyama S Common variable immunodeficiency classification by quantifying T-cell receptor and immunoglobulin κ-deleting recombination excision circles J Allergy Clin Immunolin press 10.1016/j.jaci.2012.10.059 23273952

[B43] Amariglio N Lev A Simon A Rosenthal E Spirer Z Efrati O Broides A Rechavi G Somech R Molecular assessment of thymus capabilities in the evaluation of T-cell immunodeficiency Pediatr Res 2010 67 211 216 10.1203/PDR.0b013e3181c6e554 19858778

[B44] Warnatz K Wehr C Dräger R Schmidt S Eibel H Schlesier M Peter HH Expansion of CD19(hi)CD21(lo/neg) B cells in common variable immunodeficiency (CVID) patients with autoimmune cytopenia Immunobiology 2002 206 502 513 10.1078/0171-2985-00198 12607725

[B45] Guazzi V Aiuti F Mezzaroma I Mazzetta F Andolfi G Mortellaro A Pierdominici M Fantini R Marziali M Aiuti A Assessment of thymic output in common variable immunodeficiency patients by evaluation of T cell receptor excision circles Clin Exp Immunol 2002 129 346 353 10.1046/j.1365-2249.2002.01893.x 12165093 PMC1906453

[B46] Moratto D Gulino AV Fontana S Mori L Pirovano S Soresina A Meini A Imberti L Notarangelo LD Plebani A Badolato R Combined decrease of defined B and T cell subsets in a group of common variable immunodeficiency patients Clin Immunol 2006 121 203 214 10.1016/j.clim.2006.07.003 16962827

[B47] Conley ME Rohrer J Minegishi Y X-linked agammaglobulinemia Clin Rev Allergy Immunol 2000 19 183 204 Review 10.1385/CRIAI:19:2:183 11107501

[B48] Kanegane H Futatani T Wang Y Nomura K Shinozaki K Matsukura H Kubota T Tsukada S Miyawaki T Clinical and mutational characteristics of X-linked agammaglobulinemia and its carrier identified by flow cytometric assessment combined with genetic analysis J Allergy Clin Immunol 2001 108 1012 1020 10.1067/mai.2001.120133 11742281

[B49] Hazenberg MD Borghans JA de Boer RJ Miedema F Thymic output: a bad TREC record Nat Immunol 2003 4 97 99 Review 10.1038/ni0203-97 12555089

[B50] Hatzakis A Touloumi G Karanicolas R Karafoulidou A Mandalaki T Anastassopoulou C Zhang L Goedert JJ Ho DD Kostrikis LG Effect of recent thymic emigrants on progression of HIV-1 disease Lancet 2000 355 599 604 10.1016/S0140-6736(99)10311-8 10696979

[B51] Quiros-Roldan E Serana F Chiarini M Zanotti C Sottini A Gotti D Torti C Caimi L Imberti L Effects of combined antiretroviral therapy on B- and T-cell release from production sites in long-term treated HIV-1+ patients J Transl Med 2012 10 94 10.1186/1479-5876-10-94 22591651 PMC3481359

[B52] Buckley RH Schiff SE Schiff RI Markert L Williams LW Roberts JL Myers LA Ward FE Hematopoietic stem-cell transplantation for the treatment of severe combined immunodeficiency N Engl J Med 1999 340 508 516 10.1056/NEJM199902183400703 10021471

[B53] Kane L Gennery AR Crooks BN Flood TJ Abinun M Cant AJ Neonatal bone marrow transplantation for severe combined immunodeficiency Arch Dis Child Fetal Neonatal Ed 2001 85 F110 F113 10.1136/fn.85.2.F110 11517204 PMC1721317

[B54] Myers LA Patel DD Puck JM Buckley RH Hematopoietic stem cell transplantation for severe combined immunodeficiency in the neonatal period leads to superior thymic output and improved survival Blood 2002 99 872 878 10.1182/blood.V99.3.872 11806989

[B55] Buckley RH Transplantation of hematopoietic stem cells in human severe combined immunodeficiency: longterm outcomes Immunol Res 2011 49 25 43 Review 10.1007/s12026-010-8191-9 21116871 PMC3798033

[B56] Hershfield MS Buckley RH Greenberg ML Melton AL Schiff R Hatem C Kurtzberg J Markert ML Kobayashi RH Kobayashi AL Abuchowski A Treatment of adenosine deaminase deficiency with polyethylene glycol-modified adenosine deaminase N Engl J Med 1987 316 589 596 10.1056/NEJM198703053161005 3807953

[B57] Aiuti A Cattaneo F Galimberti S Benninghoff U Cassani B Callegaro L Scaramuzza S Andolfi G Mirolo M Brigida I Tabucchi A Carlucci F Eibl M Aker M Slavin S Al-Mousa H Al Ghonaium A Ferster A Duppenthaler A Notarangelo L Wintergerst U Buckley RH Bregni M Marktel S Valsecchi MG Rossi P Ciceri F Miniero R Bordignon C Roncarolo Gene therapy for immunodeficiency due to adenosine deaminase deficiency N Engl J Med 2009 360 447 458 10.1056/NEJMoa0805817 19179314

[B58] Hacein-Bey-Abina S Hauer J Lim A Picard C Wang GP Berry CC Martinache C Rieux-Laucat F Latour S Belohradsky BH Leiva L Sorensen R Debré M Casanova JL Blanche S Durandy A Bushman FD Fischer A Cavazzana-Calvo M Efficacy of gene therapy for X-linked severe combined immunodeficiency N Engl J Med 2010 363 355 364 10.1056/NEJMoa1000164 20660403 PMC2957288

[B59] Gaspar HB Cooray S Gilmour KC Parsley KL Adams S Howe SJ Al Ghonaium A Bayford J Brown L Davies EG Kinnon C Thrasher AJ Long-term persistence of a polyclonal T cell repertoire after gene therapy for X-linked severe combined immunodeficiency Sci Transl Med 2011 3 97ra79 10.1126/scitranslmed.3002715 21865537

[B60] Brown L Xu-Bayford J Allwood Z Slatter M Cant A Davies EG Veys P Gennery AR Gaspar HB Neonatal diagnosis of severe combined immunodeficiency leads to significantly improved survival outcome: the case for newborn screening Blood 2011 117 3243 3246 10.1182/blood-2010-08-300384 21273302

[B61] Puck JM SCID Newborn Screening Working Group Population-based newborn screening for severe combined immunodeficiency: steps toward implementation J Allergy Clin Immunol 2007 120 760 768 10.1016/j.jaci.2007.08.043 17931561

[B62] Wilson JM Jungner YG Principles and practice of mass screening for disease Bol Oficina Sanit Panam 1968 65 281 393 Spanish 4234760

[B63] Baker MW Grossman WJ Laessig RH Hoffman GL Brokopp CD Kurtycz DF Cogley MF Litsheim TJ Katcher ML Routes JM Development of a routine newborn screening protocol for severe combined immunodeficiency J Allergy Clin Immunol 2009 124 522 527 10.1016/j.jaci.2009.04.007 19482345

[B64] Comeau AM Hale JE Pai SY Bonilla FA Notarangelo LD Pasternack MS Meissner HC Cooper ER DeMaria A Sahai I Eaton RB Guidelines for implementation of population-based newborn screening for severe combined immunodeficiency J Inherit Metab Dis 2010 33 Suppl 2 S273 S281 20490925 10.1007/s10545-010-9103-9

[B65] Buckley RH The long quest for neonatal screening for severe combined immunodeficiency J Allergy Clin Immunol 2012 129 597 604 Review 10.1016/j.jaci.2011.12.964 22277203 PMC3294102

[B66] Borte S von Döbeln U Hammarström L Guidelines for newborn screening of primary immunodeficiency diseases Curr Opin Hematol 2013 20 48 54 10.1097/MOH.0b013e32835a9130 23108220

[B67] Kenny AB Hitzig WH Bone marrow transplantation for severe combined immunodeficiency disease. Reported from 1968 to 1977 Eur J Paediatr 1979 131 155 177 10.1007/BF00538940 38963

[B68] Fischer A Landais P Friedrich W Morgan G Gerritsen B Fasth A Porta F Griscelli C Goldman SF Levinsky R Vossen J European experience of bone- marrow transplantation for severe combined immunodeficiency Lancet 1990 336 850 854 10.1016/0140-6736(90)92348-L 1976883

[B69] Fischer A Durandy A de Villartay JP Vilmer E Le Deist F Gerota I Griscelli C HLA-haploidentical bone marrow transplantation for severe combined immunodeficiency using E rosette fractionation and cyclosporine Blood 1986 67 444 449 3510681

[B70] Guillaume T Rubinstein DB Symann M Immune reconstitution and immunotherapy after autologous hematopoietic stem cell transplantation Blood 1998 92 1471 1490 Review 9716573

[B71] Koehl U Bochennek K Zimmermann SY Lehrnbecher T Sorensen J Esser R Andreas C Kramm C Grüttner HP Falkenberg E Orth A Bader P Schwabe D Klingebiel T Immune recovery in children undergoing allogeneic stem cell transplantation: absolute CD8+CD3+ count reconstitution is associated with survival Bone Marrow Transplant 2007 39 269 278 10.1038/sj.bmt.1705584 17311085

[B72] Passweg J Baldomero H Chapuis B Leibundgut K Schanz U Gratwohl A Swiss Transplant Working Group Blood and Marrow Transplantation Board Haematopoietic stem cell transplantation in Switzerland. Report from the Swiss Transplant Working Group Blood and Marrow Transplantation (STABMT) Registry 1997–2003 Swiss Med Wkly 2006 136 50 58 16633946 10.4414/smw.2006.11286

[B73] Klingebiel T Handgretinger R Lang P Bader P Niethammer D Haploidentical transplantation for acute lymphoblastic leukemia in childhood Blood Rev 2004 18 181 192 Review 10.1016/S0268-960X(03)00063-8 15183902

[B74] Devine SM Adkins DR Khoury H Brown RA Devine SM Adkins DR Khoury H Brown RA Vij R Blum W DiPersio JF Recent advances in allogeneic hematopoietic stem-cell transplantation J Lab Clin Med 2003 141 7 32 Review 10.1067/mlc.2003.5 12518165

[B75] Schwinger W Weber-Mzell D Zois B Rojacher T Benesch M Lackner H Dornbusch HJ Sovinz P Moser A Lanzer G Schauenstein K Ofner P Handgretinger R Urban C Immune reconstitution after purified autologous and allogeneic blood stem cell transplantation compared with unmanipulated bone marrow transplantation in children Brit J Haematol 2006 135 76 84 10.1111/j.1365-2141.2006.06244.x 16925797

[B76] Isaacs JD Thiel A Stem cell transplantation for autoimmune disorders. Immune reconstitution Best Pract Res Clin Haematol 2004 17 345 358 10.1016/j.beha.2004.04.008 15302345

[B77] van Leeuwen JE van Tol MJ Joosten AM Schellekens PT van den Bergh RL Waaijer JL Oudeman-Gruber NJ van der Weijden–Ragas CP Roos MT Gerritsen EJ van den Berg H Harraldsson A Meera Khan P Vossen JM Relationship between patterns of engraftment in peripheral blood and immune reconstitution after allogeneic bone marrow transplantation for (severe) combined immunodeficiency Blood 1994 84 3936 3947 7949150

[B78] Lau YL Kwong YL Lee AC Chiu EK Ha SY Chan CF Chan V Chan TK Mixed chimerism following bone marrow transplantation for severe combined immunodeficiency: a study by DNA fingerprinting and simultaneous immunophenotyping and fluorescence in situ hybridisation Bone Marrow Transplant 1995 15 971 976 7581099

[B79] Friedrich W Goldmann SF Ebell W Blutters–Sawatzki R Gaedecke G Raghavachar A Peter HH Belohradsky B Kreth W Kubanek B Kleihauer E Severe combined immunodeficiency: treatment by bone marrow transplantation in 15 infants using HLA- haploidentical donors Eur J Pediatr 1985 144 125 130 10.1007/BF00451897 3899661

[B80] Buckley RH Schiff SE Sampson HA Schiff RI Markert ML Knutsen AP Hershfield MS Huang AT Mickey GH Ward FE Development of immunity in human primary T cell deficiency following haploidentical bone marrow stem cell transplantation J Immunol 1986 136 2398 2407 2869085

[B81] Wijnaendts L Le Deist F Griscelli C Fischer A Development of immunologic functions after bone marrow transplantation in 33 patients with severe combined immunodeficiency Blood 1989 74 2212 2219 2804359

[B82] Kook H Goldman F Padley D Giller R Rumelhart S Holida M Lee N Peters C Comito M Huling D Trigg M Reconstitution of the immune system after unrelated or partially matched T- cell depleted bone marrow transplantation in children: immunophenotypic analysis and factors affecting the speed of recovery Blood 1996 88 1089 1097 8704219

[B83] Neven B Leroy S Decaluwe H Le Deist F Picard C Moshous D Mahlaoui N Debré M Casanova JL Dal Cortivo L Madec Y Hacein-Bey-Abina S de Saint Basile G de Villartay JP Blanche S Cavazzana-Calvo M Fischer A Long-term outcome after hematopoietic stem cell transplantation of a single-center cohort of 90 patients with severe combined immunodeficiency Blood 2009 113 4114 4124 10.1182/blood-2008-09-177923 19168787

[B84] Borghans JA Bredius RG Hazenberg MD Roelofs H Jol-van der Zijde EC Heidt J Otto SA Kuijpers TW Fibbe WE Vossen JM Miedema F van Tol MJ Early determinants of long-term T-cell reconstitution after hematopoietic stem cell transplantation for severe combined immunodeficiency Blood 2006 108 763 769 10.1182/blood-2006-01-009241 16822903

[B85] Hassan A Booth C Brightwell A Allwood Z Veys P Rao K Hönig M Friedrich W Gennery A Slatter M Bredius R Finocchi A Cancrini C Aiuti A Porta F Lanfranchi A Ridella M Steward C Filipovich A Marsh R Bordon V Al-Muhsen S Al-Mousa H Alsum Z Al-Dhekri H Al Ghonaium A Speckmann C Fischer A Mahlaoui N Nichols KE Grunebaum E Al Zahrani D Roifman CM Boelens J Davies EG Cavazzana-Calvo M Notarangelo L Gaspar HB Inborn Errors Working Party of the European Group for Blood and Marrow Transplantation and European Society for Immunodeficiency Outcome of hematopoietic stem cell transplantation for adenosine deaminase-deficient severe combined immunodeficiency Blood 2012 120 3615 3624 10.1182/blood-2011-12-396879 22791287

[B86] Cavazzana-Calvo M Carlier F Le Deist F Morillon E Taupin P Gautier D Radford-Weiss I Caillat-Zucman S Neven B Blanche S Cheynier R Fischer A Hacein-Bey-Abina S Long-term T-cell reconstitution after hematopoietic stem-cell transplantation in primary T-cell-immunodeficient patients is associated with myeloid chimerism and possibly the primary disease phenotype Blood 2007 109 4575 4581 10.1182/blood-2006-07-029090 17272510

[B87] Dvorak CC Cowan MJ Hematopoietic stem cell transplantation for primary immunodeficiency disease Bone Marrow Transplant 2008 41 119 126 Review 10.1038/sj.bmt.1705890 17968328

[B88] Gaspar HB Aiuti A Porta F Candotti F Hershfield MS Notarangelo LD How I treat ADA deficiency Blood 2009 114 3524 3532 Review 10.1182/blood-2009-06-189209 19638621 PMC2766674

[B89] Hershfield MS Adenosine deaminase deficiency: clinical expression, molecular basis, and therapy Semin Hematol 1998 35 291 298 Review 9801258

[B90] Aiuti A Brigida I Ferrua F Cappelli B Chiesa R Marktel S Roncarolo MG Hematopoietic stem cell gene therapy for adenosine deaminase deficient-SCID Immunol Res 2009 44 150 159 10.1007/s12026-009-8107-8 19224139

[B91] Gaspar HB Bjorkegren E Parsley K Gilmour KC King D Sinclair J Zhang F Giannakopoulos A Adams S Fairbanks LD Gaspar J Henderson L Xu-Bayford JH Davies EG Veys PA Kinnon C Thrasher AJ Successful reconstitution of immunity in ADA-SCID by stem cell gene therapy following cessation of PEG-ADA and use of mild preconditioning Mol Ther 2006 14 505 513 10.1016/j.ymthe.2006.06.007 16905365

[B92] Lev A Simon AJ Bareket M Bielorai B Hutt D Amariglio N Rechavi G Somech R The kinetics of early T and B cell immune recovery after bone marrow transplantation in RAG-2-deficient SCID patients PLoS One 2012 7 e30494 10.1371/journal.pone.0030494 22295088 PMC3266259

[B93] Nobile M Correa R Borghans JA D’Agostino C Schneider P De Boer RJ Pantaleo G Swiss HIV Cohort Study De novo T-cell generation in patients at different ages and stages of HIV-1 disease Blood 2004 104 470 477 10.1182/blood-2003-12-4265 15059846

[B94] Ometto L De Forni D Patiri F Trouplin V Mammano F Giacomet V Giaquinto C Douek D Koup R De Rossi A Immune reconstitution in HIV-1-infected children on antiretroviral therapy: role of thymic output and viral fitness AIDS 2002 16 839 849 10.1097/00002030-200204120-00003 11919485

[B95] De Rossi A Walker AS Klein N De Forni D King D Gibb DM Increased thymic output after initiation of antiretroviral therapy in human immunodeficiency virus type 1-infected children in the Paediatric European Network for Treatment of AIDS (PENTA) 5 Trial J Infect Dis 2002 186 312 320 10.1086/341657 12134227

[B96] Chavan S Bennuri B Kharbanda M Chandrasekaran A Bakshi S Pahwa S Evaluation of T cell receptor gene rearrangement excision circles after antiretroviral therapy in children infected with human immunodeficiency virus J Infect Dis 2001 183 1445 1454 10.1086/320197 11329124

[B97] Resino S Seoane E Pérez A Ruiz-Mateos E Leal M Muñoz-Fernández MA Different profiles of immune reconstitution in children and adults with HIV-infection after highly active antiretroviral therapy BMC Infect Dis 2006 6 112 10.1186/1471-2334-6-112 16839416 PMC1534048

[B98] Anselmi A Vendrame D Rampon O Giaquinto C Zanchetta M De Rossi A Immune reconstitution in human immunodeficiency virus type 1-infected children with different virological responses to anti-retroviral therapy Clin Exp Immunol 2007 150 442 450 10.1111/j.1365-2249.2007.03526.x 17956580 PMC2219365

[B99] Benicchi T Ghidini C Re A Cattaneo C Casari S Caimi L Rossi G Imberti L T-cell immune reconstitution after hematopoietic stem cell transplantation for HIV-associated lymphoma Transplantation 2005 80 673 682 10.1097/01.tp.0000168490.29862.b8 16177644

[B100] Resino S Pérez A Seoane E Serrano D Berenguer J Balsalobre P Goméz-Chacon GF Díez-Martin JL Muñoz-Fernández MA Short communication: Immune reconstitution after autologous peripheral blood stem cell transplantation in HIV-infected patients: might be better than expected? AIDS Res Hum Retroviruses 2007 23 543 548 10.1089/aid.2006.0071 17506611

[B101] Hansen BR Kolte L Haugaard SB Dirksen C Jensen FK Ryder LP Sørensen AL Flyvbjerg A Nielsen SD Andersen O Improved thymic index, density and output in HIV-infected patients following low-dose growth hormone therapy: a placebo controlled study AIDS 2009 23 2123 2131 10.1097/QAD.0b013e3283303307 19625946

[B102] Lorenzi AR Patterson AM Pratt A Jefferson M Chapman CE Ponchel F Isaacs JD Determination of thymic function directly from peripheral blood: a validated modification to an established method J Immunol Methods 2008 31 185 194 18854192 10.1016/j.jim.2008.09.013 PMC2593795

[B103] Baker MW Laessig RH Katcher ML Routes JM Grossman WJ Verbsky J Kurtycz DF Brokopp CD Implementing routine testing for severe combined immunodeficiency within Wisconsin's newborn screening program Public Health Rep 2010 125 Suppl 2 88 95 20518449 10.1177/00333549101250S211 PMC2846807

[B104] Ram G Chinen J Infections and immunodeficiency in Down syndrome Clin Exp Immunol 2011 164 9 16 10.1111/j.1365-2249.2011.04335.x 21352207 PMC3074212

